# An integrated multifocal tDCS-EEG protocol for reducing cognitive and affective symptoms in mild cognitive impairment and early stages of dementia: a crossover double-blind randomized controlled trial

**DOI:** 10.3389/fneur.2025.1605970

**Published:** 2025-06-18

**Authors:** Laura Catalano, Laura Sagliano, Anna Visciglio, Pasquale Russo, Stefania Miniello, Luigi Trojano, Francesco Panico

**Affiliations:** ^1^Department of Psychology, University of Campania “Luigi Vanvitelli”, Caserta, Italy; ^2^Alma Mater, Camaldoli Hospital, Naples, Italy; ^3^Neurology-Stroke Unit, AORN 'Sant'Anna e San Sebastiano' Hospital, Caserta, Italy

**Keywords:** mild cognitive impairment, early-stage dementia, non invasive brain stimulation, electrophysiology, cognitive reserve

## Abstract

**Background:**

Available evidence on effectiveness of non-pharmacological interventions for cognitive and affective symptoms in Mild Cognitive Impairment (MCI) and the early stages of dementia is encouraging but still limited. Multifocal high-definition brain stimulation could detain the potential of improving these symptoms by modulating the activity of a fronto-temporal network. Moreover, combined electrophysiological measures might contribute monitoring the underlying neurophysiological effects. In this study protocol an innovative and integrated intervention for patients with MCI and early-stage dementia will be proposed, also exploring the modulatory role of some specific variables such as education and cognitive reserve.

**Method:**

Sixty patients with MCI and early-stage dementia will be enrolled in a crossover double-blind randomized controlled trial utilizing an integrated intervention combining conventional cognitive treatment with multifocal brain stimulation and electrophysiological recordings. A battery of standardized neuropsychological tests will be employed at several time points to monitor changes, and inferential statistics will identify the changes specifically associated with the intervention. Regression analyses will be performed to ascertain the extent to which education and cognitive reserve scores may influence intervention outcomes. Analysis of electrophysiological data will contribute characterizing responders to treatment.

**Discussion:**

The project will contribute to a transformation in the landscape of non-pharmacological interventions for MCI and dementia, integrating diverse techniques and levels of analysis within a unified, comprehensive approach.

**Trial registration:**

This study is registered at ClinicalTrials.gov database under registry number NCT06668610 on October 30, 2024.

## 1 Background

As life expectancy increases and the population ages, the global prevalence of dementia is expected to continue to rise. It has been recently estimated that the number of people with dementia worldwide will increase from around 57 million cases in 2019 to 152 million cases in 2050 (1). The surge in the number of people living with dementia highlights the need for effective prevention and treatment strategies (2, 3). Pharmacological interventions have shown potential benefits in reducing cognitive decline in early dementia (4), but their overall effectiveness remains limited and warrants future advances. For this reason, in recent years the benefits of non-pharmacological interventions, such as exercise training, computerized cognitive training and cognitive stimulation, are currently being explored in dementia and its preclinical conditions (5). Within this context, non-invasive brain stimulation (NIBS), encompassing repetitive transcranial magnetic stimulation (rTMS) and transcranial direct current stimulation (tDCS), represents a safe and straightforward approach to modulating brain excitability and plasticity, with the potential to enhance cognitive functioning in individuals with dementia and Mild Cognitive Impairment (MCI). In particular, tDCS has the advantage of being highly portable, relatively non expensive and easy to use, thus being suitable even for development of telerehabilitation systems controlled by clinicians remotely (6).

Several systematic reviews and meta-analyses, however, have not provided conclusive data about effectiveness of NIBS delivered alone or combined with cognitive treatment on dementia and MCI. Teselink et al. (7) have found NIBS to significantly improve global cognition and affective symptoms relative to sham stimulation in patients with Alzheimer’s Disease (AD) and MCI. Subgroup analyses showed that these effects were mainly restricted to TMS and to patients with AD, while limited evidence was available for tDCS and MCI. Similar results were obtained by Simko et al. (8) who argued that TMS over the dorsolateral prefrontal cortex seemed to be effective in enhancing cognition in AD, whereas no clear effect was observed for MCI. In both studies no support was offered for combined cognitive stimulation therapy with NIBS intervention. A lack of relevant effects of tDCS in MCI was also confirmed by Saleh et al. (9), who called for implementation of neurophysiological methods to assess the possible brain mechanisms associated with the treatment and to identify possible markers of effective response to stimulation thus reducing variability of stimulation effects (10). These conclusions contrast with a recent systematic review and metanalysis by Yang et al. (11), according to which TMS in AD showed the best outcomes in improving global cognition, but relevant effects can be exerted by tDCS in AD and MCI as well. Crucially, Yang et al. supported a beneficial effect of NIBS combined with cognitive therapy.

Inconsistencies among the research reports could be ascribed to several factors including use of different methods to assess cognitive functions; moreover, evidence about effectiveness of tDCS in MCI is limited by the small number of studies targeting this issue, whereas more data are available on use of TMS in AD.

The objective of the present paper is to describe a protocol for the treatment of the cognitive and affective symptoms in MCI and dementia with several specific novel features. First, the protocol adopts multifocal stimulation of relevant brain structures thought to be involved in the genesis of the symptoms. While all previous studies focused on specific areas in the frontal and temporal cortex in separate treatment conditions, in the present project multifocal tDCS over such structures is proposed to the aim of maximizing distribution of direct current over the left fronto-temporal network (12). This specific feature follows recent studies describing a fronto-parieto-temporal network involved in cognitive (dys)functions which has been shown to be effectively modulated by brain stimulation (13, 14). Multifocal stimulation of such a network could strengthen the achievable outcomes by activating a larger scale network involved in cognitive and affective processes as compared to previous studies. Second, to address the issue of heterogeneity of stimulation effects, the proposed protocol combines electrophysiological measures (EEG) with brain stimulation (9). As shown in previous studies on healthy and brain damaged individuals, electrophysiological and neuroimaging techniques may contribute clarifying the mechanisms and brain response associated with brain stimulation (10, 15–18), also accounting for possible individual differences in response to stimulation. Crucially such integration would allow identifying good and bad responders to stimulation so to target individuals which may benefit the most from a given intervention (10). Third, this study protocol will further explore the variability in response to brain stimulation raised by previous studies, considering the relationship between some demographical and psychological factors with clinical and electrophysiological outcomes. Previous studies have shown a buffering effect of age on global cognitive response following brain stimulation interventions, with larger effects in younger populations (7), and a relevant association of cognitive functioning with cognitive reserve (and its proxies), as higher cognitive performance and better emotional response were associated with higher levels of cognitive reserve (3, 19). These variables will be embedded in the study protocol to allow a comprehensive interpretation of either positive or null effects, and to plan future research on the topic.

To achieve these purposes the present study protocol will adopt a double-blinded sham-controlled crossover experimental design in patients with MCI and early stages of dementia, combining brain stimulation and electrophysiological recording. The primary outcomes will be global cognitive functioning, anterograde verbal episodic memory and executive functions as well as affective symptoms. Secondary outcomes will be the level of functional independence in daily living and electrophysiological measures. Based on the available literature we expected to observe an effect of brain stimulation on cognitive and affective functions (7, 11). Moreover, we expect the use of EEG recording being able to provide findings useful to distinguish good and bad responders to the treatment based on individual analysis (9, 10). Finally, we hypothesize that demographic factors can modulate the possible outcomes, thus contributing to identify the patients most suitable for such intervention (3, 7).

## 2 Method

### 2.1 Study design

**Figure 1:**
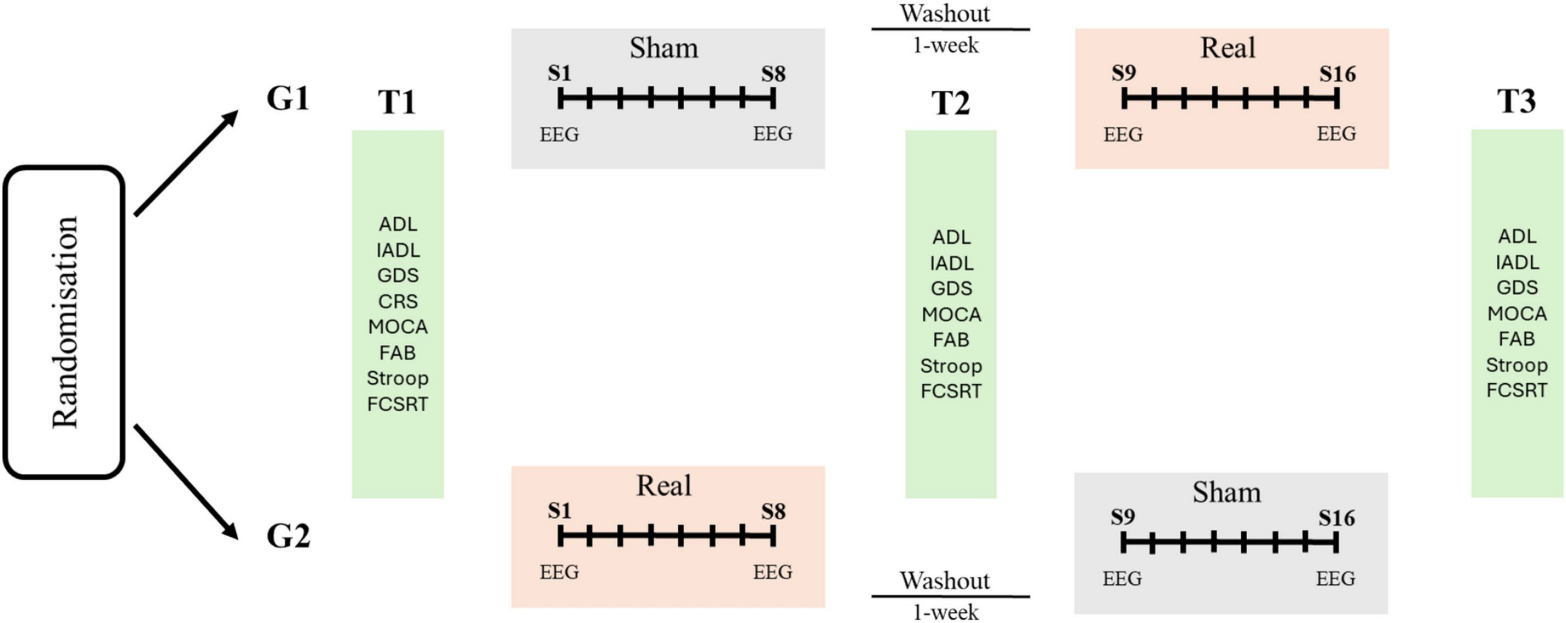
Overview and schedule for the study protocol. Participants will be randomized in two groups receiving two cycles of sham/real brain stimulation (G1) or real/sham stimulation (G2) with 1-week washout combined with cognitive therapy. Each cycle will involve eight sessions (S1–S8, S9–S16) twice-weekly. Neuropsychological assessment will be performed before and after each intervention (T1–T3). ADL: Activities of Daily Living; IADL: Instrumental Activities of Daily Living. CGS: Geriatric Depression Scale; CRS: Cognitive Reserve Scale; MOCA: Montreal Cognitive Assessment; FAB: Frontal Assessment Battery; FCSRT: Free and Cued Selective Reminding Test.

The study will adopt a double-blinded crossover randomized controlled design involving two clinical centers in Italy. The whole intervention will consist of two treatment phases (sham vs. real multifocal stimulation) and multiple assessment phases (T1, T2, T3). One-week washout will be interleaved between the two treatment phases (see Figure 1).

### 2.2 Participants

The recruitment centers for the study will be “Alma Mater Camaldoli Hospital” (Naples) and the Neuropsychological Testing Service of the Department of Psychology of University of Campania “Luigi Vanvitelli” (Caserta) in Italy. All participants consecutively admitted to the two centers will be screened for study participation. Data collection will be terminated when at least 60 participants will complete the whole intervention. Inclusion criteria are: (i) age between 55 and 85 years, and (ii) diagnosis of minor neurocognitive disorder, or major neurocognitive disorder with mild severity, according to Diagnostic and Statistical Manual of Mental Disorders [DSM-5; (20)] with a Clinical Dementia Rating Scale (CDR) of 0.05 or 1 (21), (iii) right handedness. The diagnosis will be made by expert neurologists and neuropsychologists. Exclusion criteria are: (i) brain events with an acute aetiology (stroke, traumatic brain injury, neoplastic ablation), (ii) psychiatric disorders (schizophrenia, psychosis, bipolar disorder) and assumption of psychotropic drugs, (iii) diagnosis of moderate or severe major neurocognitive disorders [DSM-5; (20)] with CDR scores equal or above 2 (21), (iv) any condition which might even hypothetically interfere with electrophysiological recording and neurostimulation (metallic implants in the brain, cochlear implant, pacemakers, epilepsy) (22, 23).

### 2.3 Procedure

The participants will be initially informed about the objectives of the study in an introductory meeting involving also their family members. Following this meeting, the participants will provide their informed consent. No compensation or different treatment within the clinical institution practices will be reserved to participants included in the study. Participants will then undergo a neuropsychological battery exploring cognitive functions, affective symptoms and independence in daily living (T1). The same battery of tests will be repeated at the end of the first (T2) and of the second (T3) rehabilitation cycles. The intervention will involve two rehabilitation periods involving multifocal brain stimulation (real vs. sham with a crossover design) combined with cognitive stimulation therapy. EEG recordings with eyes-opened will be performed at the beginning and the end of each period of stimulation. The CONSORT Flow Diagram that will be ensued detailing enrolment, allocation, follow-up, and analyses is provided as Supplementary File.

### 2.4 Intervention

The intervention will consist of multifocal brain stimulation combined with cognitive training. In a cross-over design, 16 stimulation sessions (two sessions for week; freely scheduled from Monday to Friday on participants’ and institutional needs) will be delivered in two blocks (8 real and 8 sham stimulation sessions) for 10 weeks. The first and the last stimulation sessions of each block (namely sessions 1, 8, 9 and 16; Figure 1) will be preceded and followed by 5-min resting state EEG recordings with eyes-opened. Recruited participants will be randomly assigned to a “SHAM-REAL” stimulation group (G1) or a “REAL-SHAM” stimulation group (G2; Figure 1) by computer-generated random numbers; one investigator (FP) will manage generation of allocation sequence and participants’ assignment to the groups. The experimenters responsible for delivering the stimulation (LC) will be blind to the stimulation being delivered during each session. Throughout the study, all participants will undergo traditional cognitive stimulation therapy, embracing within each session paper-and-pencil and computerized activities during a face-to-face interaction with the therapist at the rehabilitation service. Activities will target memory, attention, and executive functions (24, 25); the level of difficulty will be tailored on the patients’ neuropsychological profile and progressively adjusted based on patients’ response to the treatment. Each session of cognitive training will last 40 min and will follow the brain stimulation session.

### 2.5 Neuropsychological assessment

A brief battery of neuropsychological tests will be used to monitor cognitive functions, affective symptoms and independence in the participants involved in the treatment (T1, T2, T3; Figure 1).

*Montreal Cognitive Assessment* [MOCA; (26)]. MOCA is a brief neuropsychological tool for screening global cognitive functioning specifically designed for MCI and early stages of dementia. The test includes 8 sub-tests assessing: executive functions, visuo-spatial abilities, naming, short-term and long-term episodic memory, attention, lexical access, abstraction, spatial and temporal orientation. The test provides a maximum score of 30; the higher the score, the higher the level of global cognitive efficiency.

*Frontal Assessment Battery* [FAB; (27)]. FAB is a screening neuropsychological battery including 6 sub-tests for evaluation of executive functioning: classification, mental flexibility, motor programming, sensibility to interference, inhibitory control and environmental autonomy. The total score is up to 18; the higher the score, the higher the level of executive functioning.

*Free and Cued Selective Reminding Test* [FCSRT; (28)]. FCSRT is designed to assess episodic memory domain. The test entails learning 12 pictorial stimuli (6 living and 6 non-living), followed by free and cued recall tasks. It assesses immediate and delayed recall, both free and cued, as well as recognition ability. The FCSRT is sensitive to memory impairments where recall does not significantly improve with cues, rendering it a valuable tool for identifying early signs of the disease. The test provides separate scores for free, cued and total *immediate* recall (maximum score 36), and scores for free, cued and total *delayed* recall (maximum score 12). For these measures the higher the scores, the higher the memory performance. Moreover, the FCRS provides a cue-sensitivity index (range 0–1) representing the extent of facilitation using the semantic cues in memory recall.

*Stroop Color and Word Test* (29). The Stroop Test aims at assessing the ability to inhibit cognitive interference. Participants are presented with three tables and are required to respond as quickly as possible. Two tables represent the ‘congruent condition’: in the former participants have to read color names (hereafter referred to as color words) printed in black ink, and in the latter, they are required to name color patches. Conversely, in the third table, the so-called color-word condition, the color words are printed in an incongruent color (for example, the word ‘red’ is printed in green ink), and participants have to name the color of the ink instead of reading the word. The test provides two measures, i.e., overall number of errors and time to complete the test, with higher scores indicating worse performance.

*Geriatric Depression Scale* [GDS; (30)]. GDS is a specific evaluation tool to diagnose severity of depression symptoms in older adults. This questionnaire, including 30 items, is designed for the older person and defines his/her degree of satisfaction, quality of life, and feelings. The maximum score is 30 with higher scores indicating higher levels of depression.

*Cognitive Reserve Scale* [(31); CRS; (32)]. The Italian version of the CRS is a self-rated questionnaire evaluating the engagement of a person in several activities, i.e., daily activities, training or information, hobbies, and social life. The CRS allows to assess CR in three life stages: young adulthood (18–35 years), middle adulthood (36–64 years), and late adulthood (≥65 years). According to their ages, participants have to complete the questionnaire once, twice or three times referring to the main activities during young adulthood, middle adulthood and late adulthood. Each of the 24 items is scored on a Likert scale based on the frequency each activity is performed during a week (0 = never; 4 = twice or three-time a week). The total score of the CRS is obtained by the sum of each item (maximum score 96). The higher the score, the higher the level of CR. Depending on the age of participants the scores is averaged for each of the age period.

*Independence in Everyday Life*. To monitor functional independence two scales will be adopted. The Activities of Daily Living [ADL; (33)] scale targets activities concerning care of one’s own body, such as bathing, toileting, dressing and eating; it includes six items with a maximum score of 6. The Instrumental Activities of Daily Living [IADL; (33)] scale refers to activities to support daily life within the home and community, such as financial management, housekeeping, shopping for groceries, making telephone calls, and taking medication; it includes eight items with a maximum score of 8. Both scales will be administered to patients’ caregivers. For both scales, the higher the score the higher the level of functional independence.

### 2.6 Hybrid EEG and HD-tDCS montage

A hybrid device will be employed to record EEG and deliver anodal tDCS (StarStim 32, Neuroelectrics, Spain); the stimulation/EEG sessions will be managed by the NIC2 software v2.0.11 (Neuroelectrics, Spain).

**Figure 2:**
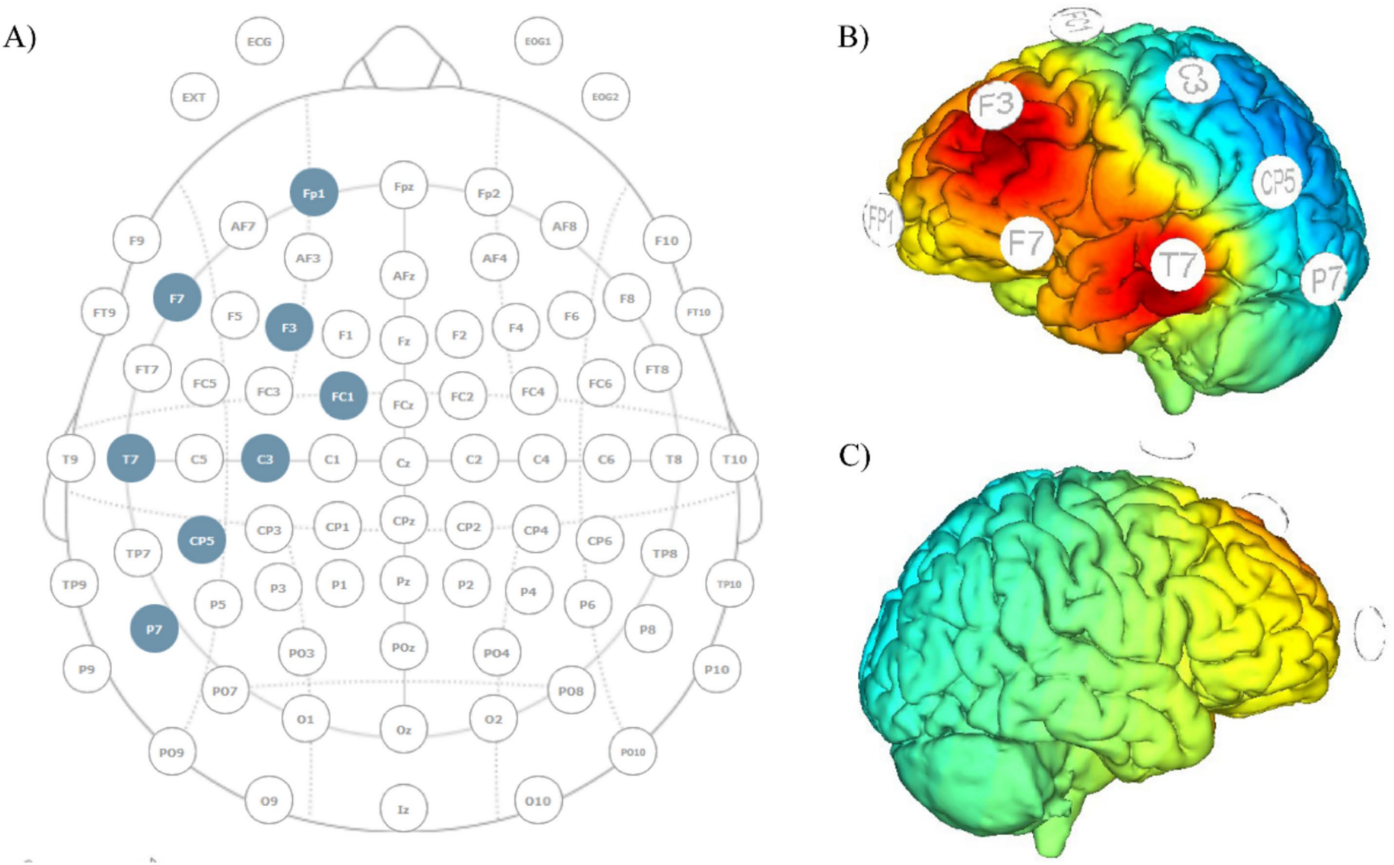
Montage template and current distribution maps. **(A)** EEG and tDCS electrodes’ placement template; anodes placed over F3 and T7, return current distributed over the remaining electrodes; **(B)** current diffusion map of the left hemisphere; **(C)** current diffusion map of the right hemisphere.

The configuration will include 8 electrodes positioned in a non-conductive neoprene cap. Direct current will be delivered using small, round gel electrodes (Ag/AgCl, 3.14 cm2), each filled with conductive electrolyte gel and positioned according to the international 10/10 EEG system. Anodal direct current delivered will be set at a total intensity of 2 mA equally distributed to the stimulation electrodes placed over F3 and T7 (1 mA each). Six return electrodes will be placed around the anodal electrodes over Fp1, FC1, F7, C3, CP5 and P7 (Figure 2) and the overall return current (−2 mA) will be equally distributed among these electrodes. This distribution of electrical current makes the minimal cathodal effects induced at the return positions not able to lead relevant changes in brain activity (Figure 2). Each stimulation session (real and sham) will last 21 min, including 30s fade-in and 30s fade-off periods. In the sham stimulation, the montage will be identical to that of the real stimulation, but the real anodal stimulation will be delivered for a duration of 30 s only. This montage will reproduce the same itching sensation associated with real stimulation thus allowing effective blinding (34).

The same electrodes used to deliver brain stimulation will serve for the EEG recordings derived before and after the stimulation sessions (sessions 1, 8, 9 and 16; Figure 1). The common mode sense (CMS) and the driven right leg (DLR) connections will be positioned on the right earlobe as electrical reference. Electrodes impedance for brain stimulation and recording will be checked before starting the session and monitored through its duration to ensure impedance values lower than 10 kΩ. Each EEG recording will last 5 min.

### 2.7 Preprocessing of EEG signal

Preprocessing of EEG data will be conducted with the open-source toolbox EEGLAB (35) in the MATLAB environment (MathWorks Inc., 2022, version 2014b). Data will be sampled at a frequency of 250 Hz and band-pass filtered with a passband between 0.1 Hz and 45 Hz to eliminate motion artefacts and physiological artefacts. Then, each EEG trace will be visually inspected to remove eyeblinks and saccades signals. The power spectral density (PSD) will be then calculated with MATLAB (MathWorks Inc., 2022, v. 2014b) for the alpha, beta, theta and gamma frequency bands, separately for each participant and session. Subsequently, a root square or a logarithmic transformation will be performed where necessary to normalize data (36).

### 2.8 Sample size justification and statistics

The final sample size of 60 participants is estimated based on a sample size calculation on G*Power (v. 3.1.9.7) suggesting a sample size of at least 48 participants to compare the two groups along three-time points with an effect size = 0.25, power = 0.80, *ε* = 0.75, and a Bonferroni-adjusted *α* = 0.0125 (0.05/4 primary cognitive outcomes see below) and considering a dropout rate up to 30% from previous studies (37, 38).

Demographic data will be reported using descriptive statistics. Primary outcomes will be the measures of cognitive (MOCA, FAB, FCSRT, Stoop scores) and affective (GDS scores) functioning, while independence in daily living (ADL and IADL scores) and EEG parameters will be considered as secondary outcomes. Intention-To-Treat (ITT) analyses (39), which includes all randomized participants in the groups assigned, regardless of their adherence with the entry criteria, will be adopted. ITT analysis admits noncompliance and protocol deviations and gives an unbiased estimate of the intervention effect. Missing data will be dealt with by using a mixed-effect model approach which accommodates missing data under the missing-at-random assumption (40). To assess the effect of the intervention on multiple dependent variables simultaneously, repeated-measures analyses of variance (ANOVAs) will be conducted on the primary outcomes and secondary outcomes separately with the factors Group (G1 vs. G2) and Time (T1-T3). Moreover, multiple regression models will be used to identify the factors associated with the treatment outcome, considering gender, age, education, CRS scores, and EEG power spectrum at baseline as predictors and post intervention measures of general cognitive functioning (MOCA and FAB) and functional independence measures as criteria.

To assess possible sequence or carryover effects, an exploratory comparison of treatment effects between the two randomization arms (sham first over real vs. real first over sham) will be performed.

Statistical analysis will be performed using IBM® SPSS® Advanced Statistics and R package (R Foundation). Statistical significance will be set at *p* < 0.05.

### 2.9 Monitoring of participants’ compliance, medications used during the trial, adverse effect and blinding

Researchers involved in administering each intervention sessions will constantly comply with a logbook completion to monitor participant compliance (i.e., adherence to each intervention sessions, complaints related to the treatments methods). In case of any change in the patient’s clinical timetable researcher involved in the intervention sessions will promptly inform the study principal investigators (FP, LC, and LT) to check the treatment plan together with the clinical setting demands. Psychotropic medications with serotonergic activity will not be permitted during the study, general medications (such as antibiotics, anti-inflammatory drugs, and vitamin supplements) will be allowed and recorded in the logbook for each patient. Adverse event, i.e., any unfavorable and unintended sign or symptom associated with the intervention, will be monitored and recorded during the trial and addressed adequately. To this purpose a checklist based on Brunoni et al. (41) will be filled in by all participants during the first session of each stimulation arm. Mitigation of adverse events will involve the use of a fade-in period prior reaching the maximum intensity of stimulation to reduce discomfort and the use of conductive gel to reduce skin irritation or burns. Blinding will be assessed via administering two *ad-hoc* scales, a Patients’ Informed, and an Assessors’ Informed Post-Intervention Scale, in which patients and investigators will be asked to guess the order of real and sham stimulation and to rate their confidence on a 5-point Likert scale.

### 2.10 Human ethics, consent to participate and compensation

Ethics approval has been obtained for this study from “Comitato Etico Territoriale Campania 1” (protocol n. 409, 29–11-23, n. 9/2023) and from the Institutional Ethic Committee of the Department of Psychology, University of Campania “Luigi Vanvitelli” (approval n. 30/2024). All participants enrolled in the study are required to provide a signed informed consent document; all family members and family doctors of the patients will be informed about study participation from their relative/assisted patient. No compensation is provided to patients participating to the study. All procedures will follow the ethical standards set by the Declaration of Helsinki.

### 2.11 Withdrawal criteria

Study participants will receive explicit information about the possibility to withdraw from the study at any point. Participants will be withdrawn if they suffer from any intolerable adverse effects resulting from tDCS or if they fail to attend required site visits, or they are noncompliant with or reluctant towards the study procedures. For transparency, though, data from all participants withdrawn from the study will be included in the final report. Unblinding will be possible only in case of withdrawal from the study.

## 3 Discussion

The present study will investigate the feasibility and efficacy of an advanced, integrated, multifocal tDCS-EEG stimulation protocol in conjunction with cognitive treatment for the management of cognitive and affective symptoms observed in MCI and early-stage dementia. The project will contribute to a substantial shift in the framework of available non-pharmacological interventions for such clinical pictures, integrating different techniques and levels of analysis in a single, comprehensive protocol. This will be achieved by four key strategies: firstly, by adopting a new multifocal protocol that allows the simultaneous stimulation of fronto-temporal regions non-invasively (12, 42); secondly, by integrating the protocol with other non-pharmacological interventions to potentially enhance the mechanisms at play; thirdly, by identifying potential electrophysiological markers to brain stimulation (10); fourthly, by exploring the factors that may modulate the response to the intervention (7). The implementation of these strategies will enable the profiling of patients who are most likely to respond to brain stimulation as a rehabilitative tool (43). In such an integrated approach, the use of brain stimulation combined with conventional cognitive therapy will allow to strengthen the plasticity-driven mechanisms associated with the behavioral treatment. Moreover, the combination in the same protocol of EEG recording will allow monitoring the brain response associated with the treatment itself. The project may provide valuable insights into the intricate changes that occur in functional brain networks during pathological ageing (44–47) and potentially effective treatment procedures.

It is important to note that the present protocol could represent a feasible, achievable and sustainable intervention based on available literature. Previous experimental studies using non-invasive brain stimulation to treat MCI and dementia used either weekly, twice weekly, or daily stimulation sessions along periods of 1 week up to months (8, 9). Considering that people with MCI are often in the working age period, and that when the level of function is compromised (i.e., since early stages of dementia), family members oversee caring for their relative with the subsequent need for balancing work-life requests, the mild-regime protocol proposed here would increase sustainability of the intervention in the long run. This protocol also considers that healthcare services are experiencing an increase in demands due to cognitive impairments in pathological ageing, and thus optimization of limited resources is highly desirable. In the future, the results from the present project might even contribute to design telerehabilitation interventions in which treatment session are conducted in the home setting with the participation of designated family members, potentially combined with computerized cognitive treatment monitored remotely by the healthcare providers (6).

It is also worth commenting that the present protocol adopts a fixed electrode montage, using HD-tDCS over F3 and T7, and does not incorporate anatomical or functional personalization of the stimulation delivered. Moreover, here EEG is used as a measure to assess the neurophysiological effects of the intervention and possible electrophysiological markers of treatment efficacy/susceptibility to response. As discussed above with reference to the main features of the present protocol, such fixed montage represents a practical and clinically translatable approach for this phase of research in a clinical population, but does not follow the perspective of individualized dose, target, and timing of brain stimulation interventions proposed in recent reviews [e.g., (48)] and suggested for rehabilitative purposes [e.g., (49)].

However, as discussed by recent studies in the field of closed-loop and brain state-dependent stimulation (50, 51), implementing truly individualized closed-loop therapeutic systems capable of reliably extracting complex brain states in real-time and identifying suitable biomarkers is currently a proof of concept and faces significant engineering and neurophysiological challenges. Moreover, strong and replicable evidence regarding the safe and effective modulation of cognitive and affective functions would be needed in healthy samples before these advanced prototypical approaches can be widely applied for rehabilitative purposes. The understanding of how our intervention affects brain oscillations seems to be a necessary step towards informing the development of future, potentially more personalized and state-dependent stimulation approaches in clinical populations.

It is possible that certain issues may impede the completion of the study protocol. A preliminary issue pertains to the participation of patients in the study, as some may prematurely withdraw from the study, thus failing to complete the full intervention program. This will be addressed through the provision of a comprehensive presentation of the study procedures and features to patients and their family members prior inclusion in the study; moreover, we will recruit a percentage of 25–30% more than the minimum sample size to ensure sufficient power to the analyses. A second issue concerns the multicenter nature of the study, which may necessitate the use of procedures that differ slightly from those employed at the other participating institution. To ensure the reliability and standardization of data acquisition and analysis processes, the experimenters involved in data acquisition at each study site will receive training and participate in regular peer-to-peer and supervision meetings. Lastly, a prompt evaluation of contraindications to brain stimulation by a physician with expertise in this field will prevent adverse effects and premature cessation of the intervention.

In conclusion, the study protocol described here responds to the new challenges posed by the ageing population, by devising an integrated rehabilitation treatment and by assessing multiple factors associated with its efficacy. Addressing these emerging issues will contribute to tackle a fundamental social problem, and to enhance quality of life of patients and their caregivers (52).
