# Supplementary material for: An integrated multifocal tDCS-EEG protocol for reducing cognitive and affective symptoms in mild cognitive impairment and early stages of dementia: a crossover double-blind randomized controlled trial
Source: Front Neurol. 2025 Jun 18;16:1605970. doi: 10.3389/fneur.2025.1605970 (PMC12213475; doi:10.3389/fneur.2025.1605970)
Supplement: Supplementary file 1 [file Supplementary_file_1.pdf]

Supplementary file: CONSORT 2025 Flow Diagram

Flow diagram of the progress through the phases of enrolment, intervention allocation, follow-up, and data analysis of two groups (G1: sham/real and G2: real/sham)

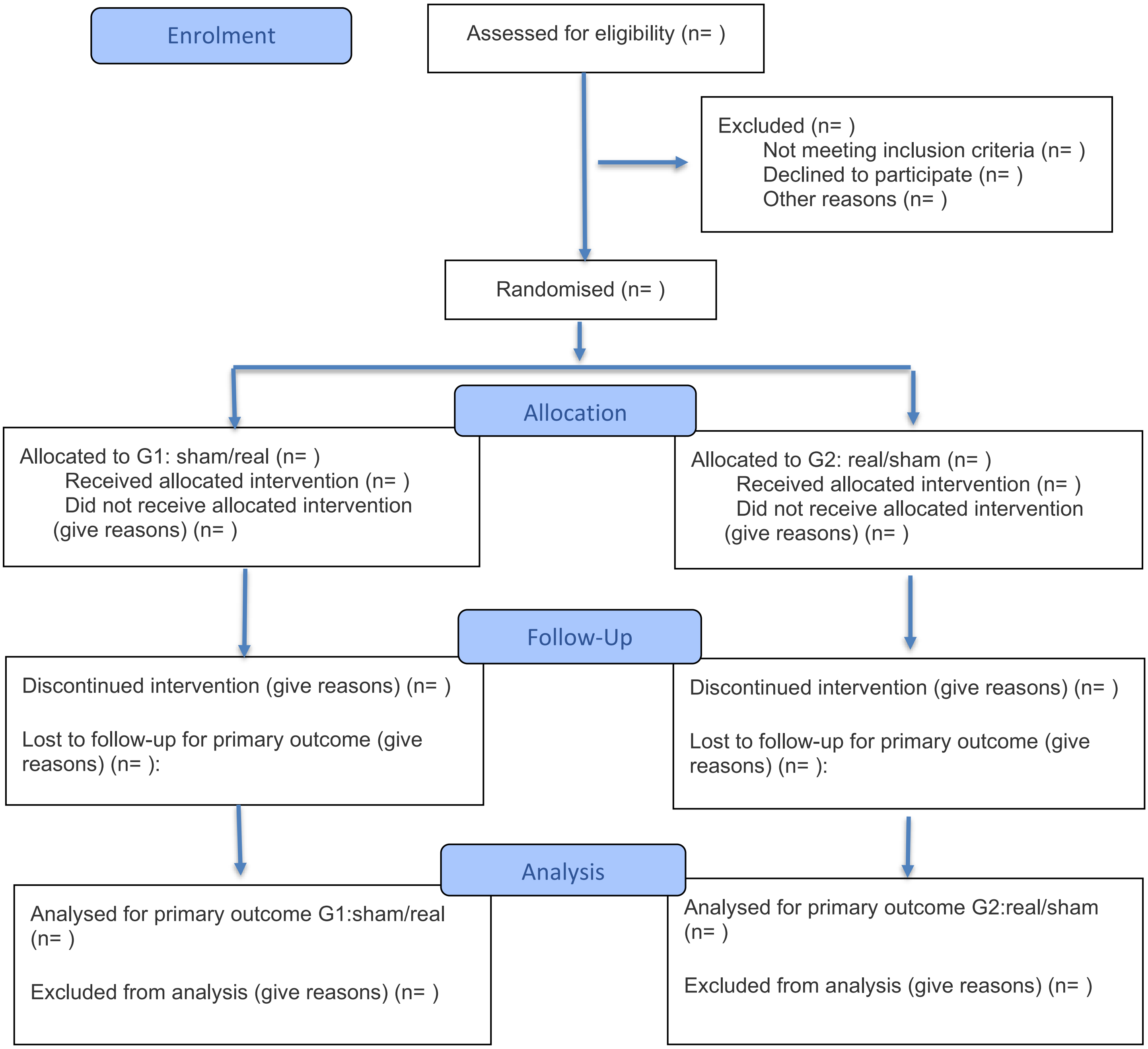

Citation: Hopewell S, Chan AW, Collins GS, Hróbjartsson A, Moher D, Schulz KF, et al. CONSORT 2025 Statement: updated guideline for reporting randomised trials. BMJ. 2025; 388:e081123. https://dx.doi.org/10.1136/bmj-2024-081123
© 2025 Hopewell et al. This is an Open Access article distributed under the terms of the Creative Commons Attribution License (https://creativecommons.org/licenses/by/4.0/), which permits unrestricted use, distribution, and reproduction in any medium, provided the original work is properly cited.
